# Clinical Marine Toxicology: A European Perspective for Clinical Toxicologists and Poison Centers

**DOI:** 10.3390/toxins5081343

**Published:** 2013-08-02

**Authors:** Corinne Schmitt, Luc de Haro

**Affiliations:** Centre Antipoison, Hôpital Sainte Marguerite, 270 Boulevard Sainte Marguerite, Marseille 13009, France; E-Mail: corinne.schmitt@ap-hm.fr

**Keywords:** jellyfish, *Physalia*, *Ostreopsis*, shellfish poisoning, lessepsian fish

## Abstract

Clinical marine toxicology is a rapidly changing area. Many of the new discoveries reported every year in Europe involve ecological disturbances—including global warming—that have induced modifications in the chorology, behavior, and toxicity of many species of venomous or poisonous aquatic life including algae, ascidians, fish and shellfish. These changes have raised a number of public issues associated, e.g., poisoning after ingestion of contaminated seafood, envenomation by fish stings, and exposure to harmful microorganism blooms. The purpose of this review of medical and scientific literature in marine toxicology is to highlight the growing challenges induced by ecological disturbances that confront clinical toxicologists during the everyday job in the European Poison Centers.

## 1. Introduction

The oceans cover most of the surface of our planet with an enormous array of biotopes. The vastness of marine biodiversity and biomass has often led man to wrongly believe that sea resources were somehow inexhaustible. Ignorance of the marine environment has also made humans easy targets for the chemical weapons that sea creatures have developed in their intense struggle for survival. Indeed, many species have developed complex defense or attack systems involving not only sting apparatus but also production of toxic molecules as weapons. Man is only starting to understand this warfare and the variety of ways that marine species use toxins. In some cases, they are injected to neutralize an enemy or prey. Some species simply store toxins in their organism and can induce intoxications when they are consumed by human beings. 

Recent advances in the field of marine toxicology have been driven by several factors. Depletion of resources in cold and temperate waters has forced fishermen who make a living from the sea to move into tropical waters to find what they can no longer have near to home. Similarly, the increase in world travel associated with globalization has had the unexpected collateral effect of confronting European physicians with previously unknown marine poisonings. The boom in exotic tourism over recent decades has been associated in an increase the risk of poisoning and envenomation for European globetrotters. Global trade has also increased the danger of exposure to marine toxins [1]. The sale of exotic foodstuffs has resulted in the occurrence of tropical food poisoning such as ciguatera and tetrodotoxism outside endemic areas. Severe envenomations have also been observed among owners of venomous marine exotic pets [2]. 

Another main factor is interest in global warming. Rising water temperatures and colonization of more and more territory by tropical species are tangible consequence of global warming [3]. Since the Mediterranean Sea is a closed sea, it is especially vulnerable to the effects of climate change and can serve as a sentinel of ecological disturbances (referred to as “tropicalization” of the Mediterranean by several authors) with the appearance of new toxic algae species and of poisonous or venomous fish during recent decades. 

The purpose of this article is to provide insight into the notion of emerging diseases in marine toxicology associated with the current ecological upheaval in the marine environment [3]. However, it must be emphasized that data in this field is changing quickly and that there are still many unknown factors. As it is impossible in one article to propose an exhaustive description of all emerging marine toxicological problems, the authors choose to give examples of problems which were at the origin of poisonings managed by clinical toxicologists in different European poison Centers. 

## 2. Blooms of Dinoflagellates, Phycotoxin, and Macroalgae

In the last few years, the whole Mediterranean has been colonized by two benthic and epiphytic algal species named *Ostreopsis cf. ovata* and *Ostreopsis siamensis* (*O. cf. ovata* is actually the main problem in the European waters and *O. siamensis* is for the moment only reported in few blooms). These two unicellular species that originate from warm waters in the Indian and Pacific Oceans are now firmly entrenched and no one quite understands how they were able to move so far from their natural biotope in such a short time [4,5]. The presence of *Ostreopsis* in the Mediterranean poses several problems. The first involves production of several toxins called ovatoxins that are all structural analogues of palytoxin. A distinctive feature of palytoxins and analogues is the ability to pass through the aquatic food chain and contaminate fish and other seafood so that it becomes unfit for human consumption [5,6]. Several reports from tropical regions have described rapidly fatal cases with diffuse severe polyvisceral complications after ingestion of fish contaminated by these toxins [7,8,9]. Recent studies suggest that the level of palytoxin-like compounds in mussels after *Ostreopsis* blooms can be hazardous for man [10]. It is therefore possible that a new form of shellfish poisonings due to contamination of filter-feeding shellfish by toxins from unicellular algae could emerge on the European coast [11,12].

Another problem caused by *Ostreopsis* algae is due to their ability to proliferate suddenly when the weather conditions are right. These proliferations are novel events since *Ostreopsis* blooms have never been reported in tropical environments. In the Mediterranean, the algae can reproduce so fast that even the water spray can become toxic for persons on the shore [13]. The most vulnerable persons are swimmers and divers who come in direct contact with the algae [14]. Recently published data obtained between 2006 and 2009 by the French network for surveillance of *Ostreopsis* blooms provides a good description of the clinical symptoms caused by skin contact or respiratory exposure to water contaminated by *Ostreopsis* [4]. Blooms similar to those observed in France have been reported from Spain, Algeria and Italy [13,15]. In Italy and Algeria, *Ostreopsis* outbreaks were at the origin of hundreds of human respiratory poisonings during recent years [4,14,15].

Algal blooms are also known to occur in fresh and brackish waters. In recent years, cyanobacteria have been implicated in several blooms resulting in animal deaths in France [16]. One such event occurred in July 2010 when two dogs died within minutes after swimming and drinking water contaminated with cyanobacterial neurotoxins not far from the City of Orange. A similar event was recorded in the spring of 2010 when several dead roe deers were found near the Tarn River following a bloom of neurotoxic cyanobacteria [3]. A recent publication described similar cases involving the death of 3 dogs and a number of sea birds by a lake following a cyanobacteria bloom in the Netherlands in the spring of 2011 [17]. These reports of rapid death by respiratory arrest in large mammals near contaminated waters underline the high risk associated with the potentially lethal toxins generated by these blooms. Regarding cyanobacteria, it is also interesting to note that several recent reports have incriminated marine cyanobacteria in food poisoning after ingestion of giant clams of the *Tridacna* genus (poisoning now referred as “ciguatera shellfish poisoning”) in New Caledonia [18] or sea turtle meat (rare marine food poisoning called Chelonitoxism) in French Polynesia [19], Micronesia and Thailand [3].

Another problem related to proliferation of algae-related is known as green tides involving the green macroalgae *Ulva lactuca*. Development of these nitrophilic macrophytes is promoted by eutrophication of the water. In France, green tides have been reported in the three departments of Brittany, *i.e.*, Morbihan, Finistere, and especially Côtes-d’Amor. Ecological data available from the bretagne-envionnement.org website are alarming. In 2009, the total surface covered by *Ulva lactuca* in the region was 20% higher than the 2002–2008 average (data for the summer of 2010 still unavailable). During 2009, nearly 90,000 m^3^ of algae was removed in 59 communities. Disposal of these algae poses a serious problem since large quantities of decomposing algae produce significant amounts of toxic gas (ammonia and hydrogen sulfide) that can be dangerous for workers [3]. This danger is well illustrated by the presence of numerous dead wild boars on the beaches in Brittany widely reported in the French press during the summer of 2011. Findings of autopsies on around 30 of these large animals indicated that the probable cause of death was acute intoxication due to hydrogen sulfide inhaled by the animals searching through the piles of macroalgae with their snouts.

In conclusion to this discussion about emerging phycotoxins, a few words should be said about the special case of ciguatera. There are a number of reasons to believe that ciguatera is an emerging poisoning threat including an increase in the number of contaminated locations, in the number of tourists affected, and in the consumers of contaminated fish. As expected, increased international travel between temperate to tropical zones has been accompanied a growing number of cases of ciguatera after ingestion of contaminated dishes during stays in endemic zones [20,21]. Upon returning home, these patients are often poorly treated by physicians with little experience with this tropical disease. Furthermore, current data indicates that the endemic zone is growing. In the Atlantic Ocean, confirmed cases of ciguatera have been reported not only in the northern hemisphere in the Canary Islands (Spain) [22,23] and Madera (Portugal) [24] but also more southerly along the African coast [25,26] posing a threat for fishing stocks used by Western countries.

## 3. Outbreaks of Cnidarians

Jellyfish outbreaks have been known for centuries and are reported regularly throughout the world. Many European countries are repeatedly confronted with sudden outbreaks, sometimes involving venomous species such as *Pelagia noctiluca* on the Mediterranean coast. These outbreaks pose a hazard for swimmers with an increase in the number and severity of envenomations (often multiple stings) in comparison with normal periods. Ecological and epidemiological data shows an increasing trend in the frequency of jellyfish outbreaks in Europe [27]. Study findings also indicate that more and more the geographical zones are affected and that the timeframe for these outbreaks is getting longer [28]. 

An outstanding illustration of this tendency is the exceptionally large outbreak of *Pelagia noctiluca* observed in Ireland in November 2007. A compact swarm of these jellyfish covering an area of 26 km^2^ to a depth of 10 meters caused several million euros in damage and resulted in the death of 100,000 salmon being raised on a fish farm [3]. The occurrence of such an outbreak so late in the year in a so northerly location is unprecedented. On the other side of the planet, a similar phenomenon involving the giant jellyfish *Nemopilema nomurai*, that can grow to a diameter of 2 meters and reach the incredible weight of 220 kg, was reported in the Northern Pacific Ocean. Since 2005, this dangerous species increased in the Sea of Japan, threatening the fishing activities on several Japanese Islands [29]. Severe envenomations involving professional fishermen have been reported in a region where cnidarians were previously rare and not considered as a significant public health issue [30].

It should be pointed out that not all cnidarian outbreaks involve poisonous animals. In Europe, large-scale beachings of the small harmless jellyfish *Velella velella* have been described on the coasts of France, Spain and Italy. Although these incidents posed no health risk, they are nevertheless witnesses of a changing ecosystem [3].

Since the middle of the 20th century, sporadic sightings of Portuguese men o’war have been reported off the Atlantic coast of France. The species involved is *Physalia physalis*, *i.e.*, a cnidarian of the Siphonophora order that is easily recognizable by its large sail-like floater under which tentacles reaching up to several meters below the surface develop. Contact with the tentacles of Portuguese man o’war can cause severe injury due to the large size of the skin lesions. The venom of Portuguese men o’war is much more toxic than that of most jellyfish and envenomation is accompanied by potentially life-threatening general symptoms (cardio- and neurotoxicity reported regularly in the tropics) [31,32]. Experience in France provides a good example of the public health issues raised by the presence of Portuguese man o’war near beach recreational areas. Since August 2008, the Poison Control Center in Bordeaux has had to deal with the recurrent presence of these animals off the Aquitaine coast during the summer months that is promptly followed by a rash of envenomation involving swimmers and surfers. A high number of envenomation can occur within a short span of time. During the summer of 2011, a total of 885 cases of envenomation by Portuguese man o’war were recorded at the Bordeaux Poison Control Center [32]. 

Another group of venomous cnidarians belongs to the *Carybdeidae* family. Stings can cause a potentially severe type of envenomation, known as Irukandji syndrome that is characterized by minor local signs but systemic disturbances with possibly life-threatening neuromuscular and cardiovascular symptoms. The strange name of this syndrome is of aboriginal origin since this type of envenomation was first described in Australia. *Carybdeidae* jellyfish species are small and difficult to identify. The first species to be incriminated in Irukandji syndrome was *Carukia barnesi* but it has been shown that stings by other species in the same family cause similar symptoms in Australia [33]. Recent publications have reported cases of Irukandji syndrome in other locations including Thailand, the USA (Florida) and the French West Indies [33,34]. 

Another cnidarian-related reaction is seabathers’ eruption that is observed at the beginning of the rainy season in the Caribbean and Gulf of Mexico. This eruption is characterized by urticarial and eczematous rash-like lesions on areas covered by the bathing suit. Lesions may be accompanied by systemic symptoms, e.g., fever, headache, muscle pain, and nausea, in the acute phase and can progress to pigmented lichenoid dermatitis or chronic granulatomatous lesions resembling prurigo. It is a skin reaction attributed to contact with the tiny larvae of cnidarian species. Known culprits include two jellyfish species, *i.e.*, *Linuche unguiculata* and *Mnemiopsis leidyi*, and one sea anemone *Edwardsiella lineata*, but other taxa may also be involved [35]. Under certain environmental conditions leading to an increase in water temperature and turbidity, these species reproduce asexually by budding and segmentation. The almost invisible larvae are less than 0.5 mm in diameter and can pass through clothing. The weight of the swimming suit then crushes the larvae against the skin causing them to release their cytotoxins. This mechanism explains that the rash appears only on covered areas. Since the year 2000, the zone in which cases are reported has expanded rapidly northward to include almost the entire Atlantic coast of Florida and southward to include most of the coast of Brazil. From an economic standpoint, this nuisance poses an impediment for the development of tourist activities in contaminated regions where swimming becomes unattractive. Several authors have implicated global warming in the recent expansion of this phenomenon [3,35].

## 4. Ascidians

A new type of poisoning involving ascidians of the *Microcosmus* genus, eaten on local seafood platters served in Southeastern France, has been recently reported in France (possible role of *Ostreopsis* blooms?) [36]. Marine animals of the same genus are also eaten in Italy and Croatia, and another species, *Pyura chilensis*, is eaten in Chile. Recent studies have demonstrated the accumulation of phycotoxins in tunicates [37]. Since January 2011, the Marseille Poison Control Centre has been consulted for several patients showing unusual symptoms after consumption of sea figs. All patients presented a similar clinical picture that was different from the classical seafood poisoning. The main findings were moderate digestive troubles and cerebellar syndrome (ataxia and dizziness) sometimes with ocular (diplopia and accommodation difficulties) and/or neuromuscular (muscle pain) symptoms that appeared 30 to 60 min after the *Microcosmus* ingestion. Samples of the sea figs involved were taken and analysis should identify the offending molecules or toxins [36].

## 5. Lessepsian and Invasive Fish Species

Lessepsian colonization is a term used to designate the movement of marine species between the Red Sea and Mediterranean Sea since the opening of the Suez Canal in 1869. The neologism “Lessepsian” used in the jargon of marine biologists comes from the name of the French engineer Ferdinand de Lesseps who was in charge of building the Suez Canal. In most cases, species originating from the Red Sea have moved into the Mediterranean Sea either under their own power for motile taxa or carried by ships in the case of sessile taxa. The colonization process is ongoing with the discovery of new invasive species every year (a total of 75 fish species between 1869 and 2009). The last three decades have witnessed an undeniable acceleration in the number of Lessepsian species [38]. 

The Lessepsian process has had consequences in the field of toxicology since a number of potentially dangerous species have been introduced. This includes the case of the jellyfish *Rhopilema nomadica* which can be at the origin of human envenomations in The Mediterranean [39]. Furthermore, two examples concerning toxic fish can be cited to illustrate this impact. The first is rabbitfish of the *Siganus* genus with two notable species, *i.e.*, *Siganus luridus* and *Siganus rivulatus*. By competing for food, these two strictly herbivorous species have practically eliminated the only local herbivorous species *Sarpa salpa*. The invaders have adapted so well to the eastern Mediterranean that they are now the most abundant species in some regions off Lebanon, Israel, and Syria. In these countries, consumption of rabittfish has become a public health issue because, like the indigenous herbivorous species *Sarpa salpa*, they can accumulate hallucinatory macroalgal toxins. This “dream” effect has been known since antiquity under the barbaric-sounding name of ichthyoallyeinotoxism [40]. Several spectacular poisoning cases attributed to *Siganus luridus* and *Siganus rivulatus* have been reported in the Middle East. Rabbitfish are doubly harmful since they also have an apparatus for envenomation with some dorsal fin spines being connected to a venom gland. Stings are painful but less intense than those of weeverfish or scorpionfish. *Siganus luridus* was first identified in the Southeastern Mediterranean Sea in 1956 and moved westward with reports from Tunisia in 1971. Suddenly and inexplicably, it crossed northward with reports in Sicily in 2004 and France in 2008 [38]. 

The second example of a poisonous Lessepsian species is the pufferfish *Lagocephalus sceleratus* that recently entered the Mediterranean Sea. Like its fugufish cousins, this tetrodon is totally unfit for consumption since its organs and skin contain lethal amounts of tetrodotoxin [1]. Fishermen in the Mediterranean are unfamiliar with this species that is regularly netted in eastern regions. Recently several severe poisonings and deaths (respiratory distress few minutes after the meal by sudden paralysis due to the brutal decrease of the neuromuscular transmission) have been reported following consumption of this highly toxic invader in Israel and Lebanon [41,42]. This fish is also present in Turkey and Greece [43,44]. Since its recent introduction, *Lagocephalus sceleratus* has already invaded the Eastern half of the Mediterranean (with proven presence in Tunisia [45] and Libya [46]) and its arrival in the western Mediterranean seems imminent [47]. It should be noted that an equally toxic indigenous pufferfish species *Lagocephalus lagocephalus* is sporadically observed along the North African Mediterranean coast from Morocco to Libya but this pelagic species from the Atlantic is rarely caught. 

The Mediterranean Sea is not the only place where invasive fish can be found. Another example involving a dangerous invasive species is the lionfish *Pterois volitans* that is a native of the Indian Ocean but was accidentally introduced from aquarium specimens into the Atlantic Ocean at the beginning of the 1990s. This venomous and carnivorous fish adapted so well to its new habitat that within the last decade it has colonized the Caribbean (presence recently reported in the Dutch and French West Indies with first lionfish envenomations reported in Martinique island in 2013 by the Marseille Poison Centre) and almost the whole eastern coast of the United States up to New England (identified in 2009). The introduction of this harmful foreign species has had a number of adverse consequences [48] on health (envenomation), ecology (predation of native species), and economy (disappearance of fishery resources).

## 6. Conclusions

Independently of the heated debate surrounding global warming, there is compelling toxicological evidence that human activity is having major ecological effects on the earth’s ecosystem with direct impact on daily activity of physicians working in seaside locations. Clinical toxicologists in European Poison Centers where such unusual patients will be managed should be prepared to deal with such emerging problems [3]. 
